# TopoTempNet: A High-Accuracy and Interpretable Decoding Method for fNIRS-Based Motor Imagery

**DOI:** 10.3390/s25175337

**Published:** 2025-08-28

**Authors:** Qiulei Han, Hongbiao Ye, Yan Sun, Ze Song, Jian Zhao, Lijuan Shi, Zhejun Kuang

**Affiliations:** 1 College of Computer Science and Technology, Changchun University, Changchun 130022, China; ye_hb1109@163.com (H.Y.); sunyan.ee@outlook.com (Y.S.); szfsy520@gmail.com (Z.S.); zhaojian@ccu.edu.cn (J.Z.); kuangzhejun@ccu.edu.cn (Z.K.); 2 Key Laboratory of Intelligent Rehabilitation and Barrier-Free Access for the Disabled, Ministry of Education, Changchun 130022, China; shilj@ccu.edu.cn; 3 Jilin Provincial Key Laboratory of Human Health State Identification and Function Enhancement, Changchun 130022, China; 4 Jilin Rehabilitation Equipment and Technology Engineering Research Center for the Disabled, Changchun 130022, China; 5 College of Electronic Information Engineering, Changchun University, Changchun 130022, China

**Keywords:** functional near-infrared spectroscopy (fNIRS), brain–computer interface (BCI), biomedical signal decoding, topological graph features

## Abstract

Functional near-infrared spectroscopy (fNIRS) offers a safe and portable signal source for brain–computer interface (BCI) applications, particularly in motor imagery (MI) decoding. However, its low sampling rate and hemodynamic delay pose challenges for temporal modeling and dynamic brain network analysis. To address these limitations in temporal dynamics, static graph modeling, and feature fusion interpretability, we propose TopoTempNet, an innovative topology-enhanced temporal network for biomedical signal decoding. TopoTempNet integrates multi-level graph features with temporal modeling through three key innovations: (1) multi-level topological feature construction using local and global functional connectivity metrics (e.g., connection strength, density, global efficiency); (2) a graph-modulated attention mechanism combining Transformer and Bi-LSTM to dynamically model key connections; and (3) a multimodal fusion strategy uniting raw signals, graph structures, and temporal representations into a high-dimensional discriminative space. Evaluated on three public fNIRS datasets (MA, WG, UFFT), TopoTempNet achieves superior accuracy (up to 90.04% ± 3.53%) and Kappa scores compared to state-of-the-art models. The ROC curves and t-SNE visualizations confirm its excellent feature discrimination and structural clarity. Furthermore, the statistical analysis of graph features reveals the model’s ability to capture task-specific functional connectivity patterns, enhancing the interpretability of decoding outcomes. TopoTempNet provides a novel pathway for building interpretable and high-performance BCI systems based on fNIRS.

## 1. Introduction

Brain–computer interface (BCI), as a cutting-edge technology enabling direct information exchange between the brain and external devices, is particularly valuable for assisting individuals with motor impairments in achieving non-muscle-dependent intent expression. Among BCI modalities, functional near-infrared spectroscopy (fNIRS) has garnered significant attention in recent research due to its non-invasiveness, portability, and excellent user adaptability [1,2]. By detecting absorption changes in near-infrared light (650–950 nm wavelength) [3], fNIRS records dynamic concentration variations in oxygenated hemoglobin (HbO) and deoxygenated hemoglobin (HbR) in the cerebral cortex, thereby indirectly reflecting hemodynamic responses evoked by neural activity [4]. Combined with the motor imagery (MI) paradigm, fNIRS-based BCI systems offer a safe and efficient neural signal decoding solution for patients with motor disabilities [5].

To further enhance decoding performance in MI tasks, researchers have progressively explored the multimodal fusion of electroencephalography (EEG) and fNIRS [6,7]. EEG provides millisecond-level temporal resolution [8], enabling real-time monitoring of neuronal population electrical activity [9], while fNIRS delivers spatial information on neurovascular coupling [10], rendering the two modalities highly complementary in spatiotemporal dimensions. For instance, Cooney et al. proposed an EEG-fNIRS hybrid BCI using dual convolutional networks for speech decoding, where concatenated EEG and fNIRS features were fed into a gated recurrent unit (GRU) layer [11]. Similarly, Arif et al. achieved cross-subject mental state recognition by fusing spatiotemporal features of EEG and fNIRS [7].

However, despite EEG’s inherent advantage in temporal resolution, its practical application faces multiple challenges. On the one hand, EEG is highly susceptible to physiological and environmental noise such as ocular/muscular artifacts and powerline interference [12,13,14], with noise control becoming particularly difficult during natural or movement conditions [15]. Wang et al. systematically verified the anti-interference advantages of fNIRS: quantitative comparisons showed superior resistance to motion and electromagnetic artifacts compared with fMRI, PET, and EEG; in practice, it is suitable for children and multi-electromagnetic environments; and in principle, it relies on near-infrared light to detect hemoglobin changes, inherently providing strong noise resistance [16].

Current fNIRS-based signal decoding methods predominantly adopt a four-stage pipeline: signal acquisition, preprocessing, feature extraction, and state classification. Feature extraction typically relies on manually designed statistical metrics including mean, variance, and slope coupled with traditional classifiers like SVM, LDA, or RF [17,18,19]. Such approaches heavily depend on handcrafted feature selection and domain expertise, constraining their generalization capability. Recently, deep learning has emerged as a mainstream technical pathway for fNIRS decoding. For example, Rojas et al. demonstrated superior performance of deep learning models such as CNN and LSTM over machine learning in pain type identification [20]. In neuroscience research, graph-based methods leverage connectivity metrics such as Pearson correlation coefficient (PCC), phase lag index (PLI), and phase locking value (PLV) to holistically analyze brain networks [21]. Representative works include Wang et al.’s fNIRSNet incorporating hemodynamic delay characteristics [22] and Cheng et al.’s approach for transforming fNIRS time series into 2D images via Gramian Angular Field fused with cognitive scale data at the decision level to improve diagnostic accuracy for amnestic mild cognitive impairment (aMCI) [23].

The inherent functional connectivity information embedded in fNIRS signals has propelled graph-based modeling into a research hotspot. To precisely capture inter-regional brain collaboration patterns, researchers have introduced learnable and dynamically updated graph structures. For instance, Seo et al. constructed PCC-based graphs with channels as nodes and inter-channel connections as edges, and then extracted functional connectivity patterns using graph convolutional networks (GCNs) [24], while Yu et al. employed coherence-based graphs with GCNs for spatiotemporal feature extraction in depression recognition [25], achieving notable improvements in decoding performance and interpretability.

Furthermore, to mitigate overfitting in fNIRS deep learning models, strategies including multimodal fusion, data augmentation, and adaptive receptive field design have been proposed [26,27,28]. Examples include the GCN-CA-CapsNet model for EEG-fNIRS emotion recognition, which fused features via Pearson correlation graphs and optimized accuracy using capsule attention mechanisms [29]. Graph theory, as a tool for modeling system elements and interactions via nodes and edges, exhibits unique advantages in brain network analysis [30]. For fNIRS channel connectivity, metrics like average degree and global efficiency have been applied to quantify functional connectivity in the dorsolateral prefrontal cortex (DLPFC) [31].

Despite these advances, critical challenges that remain are as follows: 1. Inadequate temporal modeling: most models fail to adapt to fNIRS’s low sampling rate and delayed response characteristics, limiting dynamic feature extraction. 2. Static graph structures: graph construction often relies on fixed or manual designs, unable to reflect dynamic functional connectivity during cognitive processes. 3. Weak feature fusion and interpretability: insufficient multi-scale feature integration and neural network interpretability hinder further performance gains.

To address these issues, this paper proposes TopoTempNet, a novel fNIRS-MI classification method integrating graph theory and temporal dynamic modeling, with three key contributions: 1. Multi-level graph feature modeling: constructs local channel pair and global whole-network functional connectivity graphs, extracting metrics including connection strength, density, Reciprocal of Functional Signal Mean Difference (RFSMD), and global efficiency to unify local–global brain network relationships. 2. Graph-enhanced temporal architecture: designs a hybrid temporal network combining Transformer and Bi-LSTM with graph attention to highlight critical channel pairs, capturing spatiotemporal dynamics and structural dependencies. 3. Multi-source fusion mechanism: combines raw signals, graph features, and temporal representations into a high-dimensional fusion space, boosting decoding accuracy and cross-subject generalization.

Experimental results demonstrate significant performance improvements over state-of-the-art methods on multiple fNIRS datasets, providing robust support for dynamic modeling of complex brain networks and differential region identification.

## 2. Materials and Methods

### 2.1. Selected Dataset

To evaluate the effectiveness of the proposed TopoTempNet, experiments were conducted on three publicly available datasets.

#### 2.1.1. MA Dataset [32]

This dataset includes 29 participants (mean age: 28.5 ± 3.7 years). During experiments, participants performed mental arithmetic (MA) tasks and baseline (BL) tasks. In MA tasks, participants memorized arithmetic expressions displayed on-screen and performed consecutive addition/subtraction operations. BL tasks required participants to remain motionless without additional instructions. Each trial began with a 2 s cue period, followed by a 10 s task execution phase, with 15–17 s of rest between trials. Each participant completed three experimental blocks, with 10 MA and 10 BL trials per block.

#### 2.1.2. WG Dataset [33]

Comprising 26 subjects (mean age: 26.1 ± 3.5 years), this dataset involved word generation (WG) and baseline (BL) tasks. During WG tasks, subjects generated as many words as possible starting with a displayed letter within a time limit, while BL tasks required quiet rest. Each task type was repeated 30 times, with 10 s task durations and 13–15 s of rest intervals.

#### 2.1.3. UFFT Dataset [34]

This dataset contains fNIRS signals from 30 volunteers (17 males) performing three motor tasks: right-hand tapping (RHT), left-hand tapping (LHT), and foot tapping (FT), with 25 trials per task. Each trial consisted of a 2 s cue phase, a 10 s execution phase, and 17–19 s of rest.

### 2.2. Data Preprocessing

The near-infrared spectroscopy (NIRS) acquisition system records raw optical density changes (ΔOD), which are converted to oxygenated hemoglobin (ΔHbO) and deoxygenated hemoglobin (ΔHbR) concentration changes using the modified Beer–Lambert law [35]. The mathematical formulation is as follows:

(1) $$\left[\begin{array}{c} \Delta HbO\\ \Delta HbR \end{array}\right]=\frac{{\left[\begin{array}{c} \varepsilon_{HbO\left(\lambda_{1}\right)}\varepsilon_{HbR\left(\lambda_{1}\right)}\\ \varepsilon_{HbO\left(\lambda_{2}\right)}\varepsilon_{HbR\left(\lambda_{2}\right)} \end{array}\right]}^{-1}\left[\begin{array}{c} \Delta OD\left(t,\lambda_{1}\right)\\ \Delta OD\left(t,\lambda_{2}\right) \end{array}\right]}{d\times l},$$

where $\varepsilon_{HbO\left(\cdot \right)}$ and $\varepsilon_{HbR\left(\cdot \right)}$ denote the extinction coefficients of HbO and HbR at wavelength λ, d  is the differential pathlength factor, and l represents the source–detector separation distance.

Following the protocols described in the respective dataset references, the NIRS signals for MA and WG were downsampled to 10 Hz, while UTTF maintained its original 13.3 Hz sampling rate. Since raw fNIRS signals contain instrument noise, physiological noise, and motion artifacts, task-specific filtering strategies were applied, i.e., MA and UTTF: bandpass filter (0.01–0.1 Hz); WG: lowpass filter (cutoff: 0.2 Hz).

Subsequently, signals were segmented into non-overlapping 1 s epochs. To account for hemodynamic response delays, each epoch included pre- and post-stimulus periods to ensure the complete capture of task-related dynamics. Baseline correction was performed by subtracting the mean value of the pre-stimulus reference interval from each fNIRS epoch to mitigate drift.

**Figure 1:**
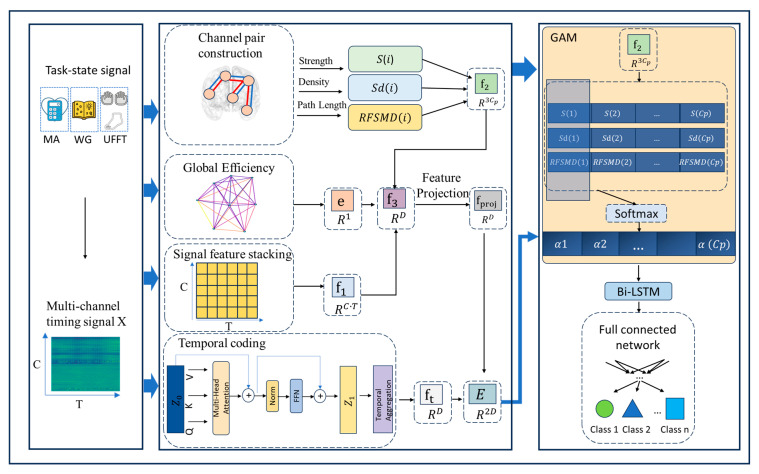
The overall architecture of the model.

As illustrated in Figure 1, the proposed TopoTempNet framework is designed for multi-class time series signal classification. Its core innovation lies in a multi-level graph-theoretic feature extraction framework that quantifies inter-channel functional connectivity patterns (local associations and global network properties), integrated with a hybrid GAM-Bi-LSTM architecture for precise temporal pattern classification.

### 2.3. Data Structure Definition

The model input data are represented as a multichannel temporal signal matrix:

(2) $$X\in R^{C\times T},$$

where C denotes the total number of channels, and T represents the number of time points in each signal segment. To further model the pairing relationships between channels, let $C=2C_{p}$, meaning that all channels are grouped into $C_{p}$ pairs, with each pair corresponding to a potential functional connection.

To construct the initial feature representation, X is stacked column-wise and reshaped into a one-dimensional vector as follows:

(3) $$f_{1}\in R^{C\cdot T},$$

This vector serves as the foundational feature representation, preserving the joint information across channels and time dimensions from the original temporal signals, thereby providing the input basis for subsequent graph-structured and temporal modeling.

### 2.4. Channel Pair Construction

To more effectively model the functional connectivity between brain regions, this study converts the raw optical density change signals using the modified Beer–Lambert law to extract the concentration changes in oxygenated hemoglobin (HbO) and deoxygenated hemoglobin (HbR). Since each measurement channel contains both HbO and HbR hemodynamic signals, this study treats them as the fundamental unit of functional coupling—referred to as a channel pair—with a total of $C_{p}$ pairs.

### 2.5. Graph-Theoretic Feature Extraction Module

This module aims to extract two types of key features from fNIRS signals through graph-theoretic analysis: local connectivity features (based on channel pairs) and global topological features (based on the overall network structure), incorporating both raw signal characteristics and functional connectivity features.

#### 2.5.1. Local Feature Extraction of Homologous Channel Pairs

For each channel pair (the i-th pair), its local functional connectivity characteristics are quantified from the following three aspects:

- Connection Strength: it is defined as the normalized Pearson correlation coefficient, measuring the linear association between the channels in the pair.

(4) $$s\left(i\right)=\frac{1}{2}\left(\rho \left(x_{i},x_{i}^{\prime }\right)+1\right),s\left(i\right)\in \left[0,1\right],$$

where $x_{i},x_{i}^{\prime }\;\in R^{T}$ represent the HbO and HbR signals of the *i*-th channel pair, respectively, and $\rho \left(\cdot \right)$ denotes the Pearson correlation coefficient:

(5) $$\rho \left(x_{i},x_{j}\right)=\frac{\sum_{t=1}^{T}\left(x_{i}\left(t\right)-\bar{x_{i}}\right)\left(x_{j}\left(t\right)-\bar{x_{j}}\right)}{\sqrt{\sum_{t=1}^{T}{\left(x_{i}\left(t\right)-\bar{x_{i}}\right)}^{2}}\sqrt{\sum_{t=1}^{T}{\left(x_{j}\left(t\right)-\bar{x_{j}}\right)}^{2}}},$$

where$\;\bar{x_{i}}=\frac{1}{T}\sum_{t=1}^{T}x_{i}\left(t\right)\;$ is the mean of $x_{i}$, and $x_{i}\left(t\right)$ denotes the value at the *t*-th time point. After normalization, $s\left(i\right)$ ranges within (0, 1), with larger values indicating stronger connectivity between the two channels.

- Connection Density: it is defined as the proportion of significant correlations within a sliding window, measuring the stability of the connection.

(6) $$Sd\left(i\right)=\frac{1}{T-L+1}\sum_{k=1}^{T-L+1}1(\rho \left|x_{i}^{\left(k\right)},{x^{\prime }}_{i}^{\left(k\right)}\right|>0.3),$$

where *L* is the length of the sliding window, $x_{i}^{\left(k\right)}=x_{i}\left(k\right),x_{i}\left(k+1\right),\ldots ,x_{i}{\left(k+L-1\right)}^{⊤}\in R^{L}\;$ represents the signal segment in the *k*-th sliding window, 0.3 is the significance threshold, and 1(⋅) is the indicator function (equals 1 if the condition is true, otherwise 0). A larger value of $Sd\left(i\right)$ indicates more frequent effective connectivity between the two channels.

- Reciprocal of Functional Signal Mean Difference (RFSMD): it is defined as the reciprocal of the mean difference between two channel signals, used to quantify the efficiency of functional information transfer between channel pairs.

(7) $$RFSMD\left(i\right)=\frac{1}{\frac{1}{T}\sum_{t=1}^{T}\left|x_{i}\left(t\right)-x_{i}^{\prime }\left(t\right)\right|+\varepsilon },$$

where $\varepsilon =10^{-6}$ is a small constant added to avoid division by zero. A smaller value indicates greater similarity between the two channel signals and higher information transfer efficiency.

#### 2.5.2. Global Network Feature Extraction

A functional connectivity graph is constructed at the full-channel scale, and topological metrics of the overall brain network are extracted.

- Construction of the Functional Connectivity Network

An adjacency matrix $A\in R^{C\times C}$ is constructed to indicate whether a significant functional connection exists between channels:

(8) $$A_{c_{1},c_{2}}=\{\begin{array}{c} 1,(\left|\rho \left(x_{c1},x_{c2}\right)\right|>0.3)\\ 0,\;\;\;otherwise \end{array},$$

where ρ(·) denotes the Pearson correlation coefficient, and 0.3 is the global connection threshold. $A_{c_{1},c_{2}}$ = 1 indicates that a significant functional connection exists between the two corresponding channels.

- Global Efficiency

It is defined as the average of the inverse shortest path lengths between all pairs of nodes in the network, and global efficiency measures the average efficiency of information transfer across the entire network. It is formally defined as follows:

(9) $$e=\frac{1}{C\left(C-1\right)}\sum_{\begin{array}{c} C_{1}=1\\ C_{1}\neq C_{2} \end{array}}^{C}\sum_{C_{2}=1}^{C}\frac{1}{d_{C_{1},C_{2}}},$$

where $d_{C_{1},C_{2}}$ denotes the shortest path length between nodes $C_{1}$ and $C_{2}$, calculated based on the adjacency matrix A. The global efficiency e∈ [0, 1], with higher values indicating more efficient global information transfer within the network.

### 2.6. Feature Fusion Module

To integrate information from multiple sources, this study designs a feature fusion mechanism that concatenates the raw temporal signal features, graph-theoretic local connectivity features, and global topological metrics into a unified representation. The details are as follows:

(10) $$f_{3}={\left[{\left(f_{1}\right)}^{⊤},{\left(f_{2}\right)}^{⊤},e\right]}^{⊤},$$

where $f_{3}\in R^{D}$, and $f_{2}={\left[s\left(1\right),Sd\left(1\right),sp\left(1\right),\ldots ,s\left(C_{p}\right),Sd\left(C_{p}\right),RFSMD\left(C_{p}\right)\right]}^{⊤}\in R^{3C_{p}}$ is the vector formed by sequentially stacking the local features of all channel pairs. The dimension of the fused feature vector is *D* = *C* $\cdot \;T+3C_{p}+1$.

### 2.7. Temporal Modeling Module

#### 2.7.1. Transformer Temporal Encoding [36]

To model the temporal dynamics of fNIRS signals, this study introduces a Transformer module to encode the channel pair signals. The specific steps are as follows:

1. Input Projection: the dual-channel signals (HbO and HbR) of each channel pair are mapped into a high-dimensional feature space:
  (11) $$Z_{0}=W_{p}X+b_{p},$$
  where $Z_{0}\in R^{C_{p}\times T\times d}$, with *d* representing the feature dimension; $W_{P}\in R^{D\times 2}$ is the projection weight matrix, and $b_{P}\in R^{D}$ is the bias vector.
2. Transformer Encoding: A standard Transformer layer is applied to model the temporal dependencies within the time series of each channel pair, capturing relationships across different time points:
  (12) $$Z_{1}=Transformer\left(Z_{0}\right)\in R^{C_{p}\times T\times D},$$
3. Temporal Aggregation: average pooling is performed along the temporal dimension to obtain an overall temporal representation for each channel pair
  (13) $$f_{t}=\frac{1}{T}\sum_{t=1}^{T}Z_{1}^{\left(t\right)},$$
  where $f_{t}\in R^{D},$ and $Z_{1}^{\left(t\right)}\in R^{C_{p}\times D}$ represents the features of $Z_{1}$ at the *t*-th time point.

#### 2.7.2. Feature Projection and Fusion

To achieve the unified alignment of features from different sources, the fused feature $f_{3}$ is projected into the same feature space dimension as the Transformer output:

(14) $$f_{proj}=W_{w}\cdot f_{3}+b_{w},$$

where $f_{\mathrm{proj}}\in R^{D}$ is the projected fused feature, $W_{w}\in R^{d\times D}$ is the projection weight matrix, and $b_{w}\in R^{D}$ is the bias vector. This is then further fused with the temporal features $f_{t}$ extracted by the Transformer to construct a unified input representation:

(15) $$E=\left[f_{t},f_{proj}\right],$$

where $E\in R^{2D}$. This process jointly represents structural (graph-theoretic) and dynamic (temporal) features on a unified scale, providing the foundation for subsequent attention modulation and decoding.

#### 2.7.3. Graph-Theoretic Temporal Association Network Module

This module integrates the topological characteristics of the functional connectivity network with temporal dynamics through graph-theory-guided gated sequence modeling. The core lies in the design of a graph attention mechanism (GAM), which enables the precise capture of dynamic relationships between channel pairs by fusing the output features of the subsequent Bi-LSTM. Specifically, GAM utilizes weights $\alpha_{i}$ derived from graph-theoretic metrics (such as connection strength) to modulate the input sequence to LSTM. By introducing graph structural priors into the gating layer, it achieves topology-aware temporal attention modeling.

Graph Attention Mechanism (GAM): It assigns differentiated semantic weights to different channel pairs. For the *i*-th channel pair, the attention weight $\alpha_{i}$ is defined as follows:

(16) $$\alpha_{i}=Softmax(W_{\alpha }\cdot [s\left(i\right),sd\left(i\right),RFSMD\left(i\right){]}^{⊤}),$$

where $W_{\alpha }\in R^{1\times 3}$ is the learnable attention weight matrix, producing the attention weights $\alpha \in R^{C_{p}}$ for the channel pairs.

#### 2.7.4. Bidirectional Temporal Association Modeling (Bi-LSTM)

**Figure 2:**
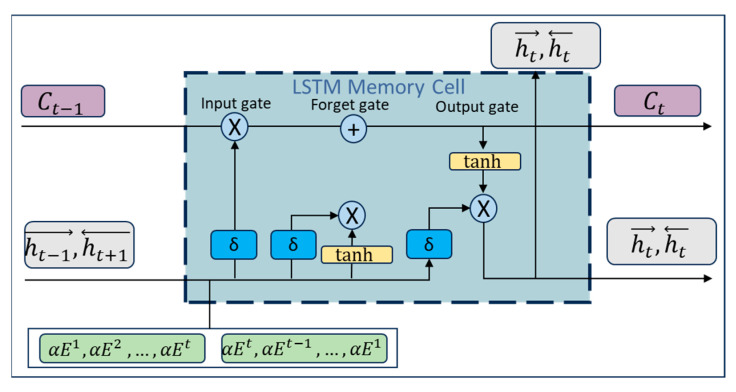
LSTM module structure.

The Bidirectional Temporal Association Modeling (Bi-LSTM) module aims to capture bidirectional temporal dependencies in the fused feature sequences using forward and backward LSTM units (as shown in Figure 2), and enhances attention to dynamic patterns of critical channel pairs by incorporating graph-theoretic attention weights.

Input Data: The input consists of the fused feature sequence E (formed by concatenating temporal features from the Transformer and projected graph-theoretic features, $E\in R^{2d}$) and the graph attention weights α (from the GAM module, $\alpha \in R^{C_{p}}$). Here, α is used to modulate the LSTM’s focus on channel pairs with strong functional connectivity.

Gating Mechanism and Equations: each LSTM unit updates its state dynamically through the forget gate, input gate, and output gate, where all gating operations incorporate the attention weights α:

Forget Gate: It determines the proportion of the historical hidden state to retain. The formula is as follows:

(17) $$g_{t}=\sigma \left(W_{g}\cdot \left[\vec{h_{t-1}},E^{\left(t\right)},\alpha \right]+b_{f}\right),$$

where $W_{g}$ is the weight matrix of the forget gate, $b_{f}$ is the bias term, σ is the sigmoid activation function, and $\vec{h_{t-1}}$ denotes the forward hidden state from the previous time step.

Input Gate: it regulates the contribution of new features to the current cell state.

(18) $$i_{t}=\sigma \left(W_{i}\cdot \left[\vec{h_{t-1}},E^{\left(t\right)},\alpha \right]+b_{i}\right),$$

where $W_{i}$ is the input gate weight matrix, and $b_{i}$ is the bias term.

Cell State Update:

(19) $$\tilde{c_{t}}=tanh\left(W_{c}\cdot \left[\vec{h_{t-1}},E^{\left(t\right)},\alpha \right]+b_{c}\right),$$

(20) $$c_{t}=g_{t}⊙C_{t-1}+i_{t}⊙\tilde{C_{t}},$$

where $W_{c}$ is the weight matrix for the cell state, $b_{c}$ is the bias term, and ⊙ denotes element-wise multiplication.

Output Gate: it generates the current hidden state.

(21) $$o_{t}=\sigma \left(W_{o}\cdot \left[\vec{h_{t-1}},E^{\left(t\right)},\alpha \right]+b_{o}\right),$$

(22) $$\vec{h_{t}}=o_{t}⊙\tan h\left(c_{t}\right),$$

where $W_{o}$ is the output gate weight matrix, $b_{o}$ is the bias term, and $\vec{h_{t}}$ is the current forward hidden state.

Bidirectional Modeling Output:

(23) $$h_{t}=\left[\vec{h_{t}},\overset{\leftarrow }{h_{t}}\right],$$

The final output is obtained by concatenating the forward and backward hidden states: $h_{t}\in R^{2h}$, where *h* is the dimension of the hidden layer.

### 2.8. Classification Module

The classification layer takes the fused features and temporal association features as inputs and outputs the classification results through a multilayer perceptron (MLP).

#### 2.8.1. Input Feature Definition

The fused features and temporal association features are concatenated and integrated through a linear transformation to form the input representation:

(24) $$f_{cls}=W_{1}\left[f_{3},h_{t}\right]+b_{1},$$

where $W_{1}\in R^{m\times \left(D+2h\right)}$ is the learnable weight matrix (with mm denoting the dimension of the intermediate layer), $b_{1}\in R^{m}$ is the bias vector, and $f_{cls}\in R^{m}$ represents the intermediate classification feature.

#### 2.8.2. Nonlinear Activation

The ReLU function is applied to enhance the nonlinear representation capability of the features:

(25) $$f_{\mathrm{act}}=\mathrm{ReLU}\left(f_{\mathrm{cls}}\right),$$

#### 2.8.3. Classification Output

The final classification probability distribution is produced via a fully connected layer followed by a Softmax function:

(26) $$\hat{y}=Softmax\left(W_{2}\cdot f_{act}+b_{2}\right),$$

where $W_{2}\in R^{K\times m}$ (with KK denoting the number of classes), $b_{2}\in R^{K}$ is the output bias, and $\hat{y}\in R^{K}$ represents the probability distribution.

## 3. Experimental

### 3.1. Experimental Setup

All experiments were conducted on a GPU server with CUDA-compatible drivers using the PyTorch (v2.7.1) framework. The model was trained with the Adam optimizer [37] (learning rate = 1 × 10^−3^, weight decay = 1 × 10^−4^). A ReduceLROnPlateau scheduler [38] adjusted the learning rate based on validation accuracy. 

Training used 5-fold cross-validation per subject, with a batch size of 5 and a maximum of 200 epochs. To prevent overfitting, dropout was applied in two modules:

- Bi-LSTM Module: a 0.1 dropout rate after temporal feature projection.
- Classifier Module: a 0.2 dropout rate in the intermediate MLP layer to enhance robustness and generalization.

### 3.2. Evaluation Metrics

Model performance was evaluated using standard classification metrics:

*Accuracy*: it measures overall prediction correctness.

(27) $$Accuary=\frac{TP+TN}{TP+TN+FP+FN},$$

ROC and AUC: the ROC curve evaluates classifier performance, with AUC indicating the model’s overall effectiveness (closer to 1 is better).

(28) $$TPR=\frac{TP}{TP+FP},$$

(29) $$FPR=\frac{TP}{FP+TN},$$

Cohen’s *Kappa*: it assesses agreement beyond chance, especially under class imbalance.

(30) $$Kappa=\frac{p_{o}-p_{e}}{1-p_{e}},$$

where

(31) $$p_{o}=\frac{TP+TN}{N},$$

(32) $$p_{e}=\frac{\left(TP+FP\right)\left(TP+FN\right)+\left(FN+TN\right)\left(FP+TN\right)}{N^{2}},$$

Higher *Kappa* values indicate more reliable and consistent classification.

## 4. Results and Discussion

**Figure 3:**
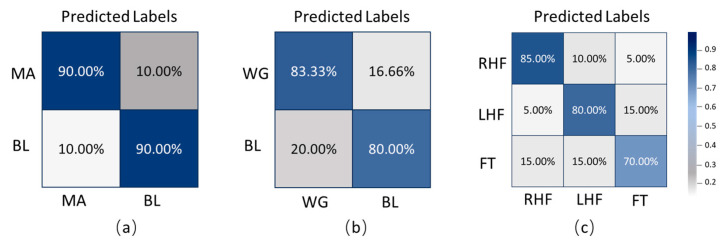
Average confusion matrices of the proposed TopoTempNet model: (**a**) MA dataset, (**b**) WG dataset, and (**c**) UFFT dataset.

Figure 3 presents the average subject-wise confusion matrices under three tasks: two binary classification tasks (mental arithmetic versus baseline, word generation versus baseline) and one three-class motor imagery task (right-hand imagery, left-hand imagery, foot imagery). In the mental arithmetic versus baseline task, an average of 90 percent of real MA samples were correctly predicted as MA, while 10 percent were misclassified as BL. Similarly, 90 percent of real BL samples were correctly recognized, with 10 percent misclassified as MA, demonstrating good classification performance for this task. In the word generation versus baseline task, 83.33 percent of real WG samples were correctly identified, while 16.66 percent were misclassified as BL. Meanwhile, 80 percent of BL samples were correctly classified, and 20 percent were misclassified as WG. For the three-class motor imagery task, 85 percent of RHF samples were correctly classified, 10 percent misclassified as LHF, and 5 percent as FT. Among the LHF samples, 80 percent were correctly identified, 5 percent misclassified as RHF, and 15 percent as FT. For FT samples, 70 percent were correctly classified, while 15 percent were misclassified as RHF and another 15 percent as LHF. These matrices intuitively illustrate the model’s average classification performance across different tasks by presenting the proportions of correct and incorrect classifications for each class.

### 4.1. Comparative Experiments

#### 4.1.1. Subject-Specific Evaluation

In this study, the proposed TopoTempNet model is systematically benchmarked against a series of structurally comparable baseline methods. To ensure rigor and reproducibility, all baseline models were faithfully re-implemented according to the hyperparameter configurations reported in their original publications. Furthermore, the entire preprocessing pipeline, input representation, and training and evaluation protocols were standardized across all models for a fair comparison.

The comparative results, encompassing both classification accuracy (Acc) and inference time (IT), are summarized in Table 1. Here, Acc denotes the predictive accuracy of the model, while IT quantifies the computational cost incurred during the inference stage. The baseline methods under evaluation are detailed as follows:

- CNN [39]: It consists of three convolutional layers and two dense layers. The input is the Δ[HbO_2_] and ΔHbR data stacked along a new dimension after separation.
- LSTM [39]: A basic three-layer Long Short-Term Memory model with a hidden size of 20. The input includes the unseparated Δ[HbO_2_] and ΔHbR signals [37].
- PVTv2-B0 [40]: A vision Transformer model using 4 × 4 patches and an eight-layer structure. Multichannel GADF-transformed virtual images are used as input.
- MLP-Mixer [40]: An eight-layer MLP-Mixer model based on 4 × 4 patches, which captures features through channel-mixing and spatial-mixing MLPs. It also takes multichannel GADF-transformed virtual images as input.
- fNIRS-T [41]: A Transformer-based classification model designed to capture long-range dependencies in fNIRS signals.
- FCS-TPNet [42]: It learns delayed hemodynamic features and captures the correlation between HbO and HbR through a dual-signal interaction module. A dynamic graph convolution module is employed to extract complex topological patterns between channels for fNIRS signal classification.

**Table 1:** Performance comparison of different models on three public datasets (bold indicates the best performance). Acc denotes model accuracy, and IT denotes inference time.

Method	MA			WG			UFFT		
	Acc (%)	Kappa	IT	Acc (%)	Kappa	IT	Acc (%)	Kappa	IT
CNN	72.23 ± 3.36	0.52	**1.70 ± 0.11**	69.73 ± 2.12	0.40	**1.66 ± 0.12**	70.02 ± 5.22	0.58	**1.69 ± 0.23**
LSTM	73.50 ± 10.74	0.61	1.84 ± 0.33	71.20 ± 7.38	0.41	1.71 ± 0.15	70.28 ± 4.16	0.58	1.70 ± 0.16
PVTv2-B0	78.26 ± 6.59	0.64	2.12 ± 0.44	72.88 ± 6.22	0.44	1.85 ± 0.23	72.08 ± 4.98	0.59	1.92 ± 0.53
MLP-MIXer	78.73 ± 6.76	0.64	3.96 ± 0.24	76.44 ± 4.36	0.58	6.54 ± 0.68	72.51 ± 3.46	0.53	2.12 ± 0.44
fNIRS-T	78.96 ± 4.38	0.65	1.90 ± 0.44	76.50 ± 4.17	0.49	1.75 ± 0.38	79.21 ± 3.44	0.64	1.95 ± 0.43
FCS-TPNet	80.02 ± 4.12	0.60	2.03 ± 0.53	76.36 ± 3.38	0.46	8.99 ± 0.45	79.26 ± 3.16	0.66	3.68 ± 0.82
**TopoTempNet (Ours)**	**90.04 ± 3.53**	**0.77**	1.91 ± 0.40	**78.33 ± 5.42**	**0.62**	1.76 ± 0.12	**81.66 ± 3.23**	**0.68**	1.91 ± 0.44

#### 4.1.2. ROC Curve Analysis of Different Models in Multi-Task fNIRS Classification

**Figure 4:**
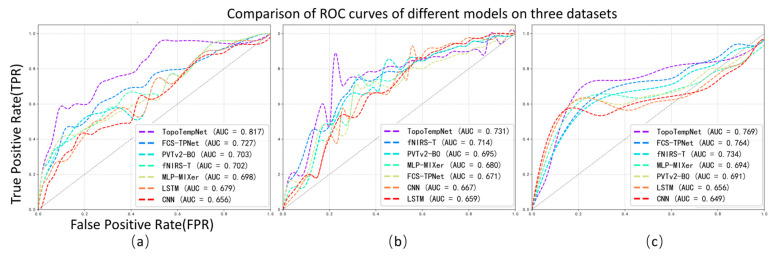
Average ROC curves and corresponding AUC values of different models across the three datasets.

In this study, we further analyzed the performance of the TopoTempNet model on three different task datasets. The horizontal axis represents the false positive rate (FPR), and the vertical axis represents the true positive rate (TPR). Different colored curves correspond to different models. The corresponding ROC curves are shown in Figure 4.

Figure 4a: The horizontal axis is the false positive rate (FPR), and the vertical axis is the true positive rate (TPR), with different colors representing various models. TopoTempNet (AUC = 0.817) stands out with its curve closer to the top-left corner, indicating stronger ability to correctly identify true positives while avoiding false positives in distinguishing brain signals related to the mental arithmetic task. Compared to other models, such as FCS-TPNet (AUC = 0.727) and fNIRS-T (AUC = 0.702), whose curves lie relatively lower with smaller AUC values, their performance in recognizing the mental arithmetic state is weaker than TopoTempNet, highlighting TopoTempNet’s advantage in classifying brain signals for this task.

Figure 4b: TopoTempNet (AUC = 0.769) again shows a curve closer to the top-left corner, with a higher AUC than other models. Models like FCS-TPNet (AUC = 0.764) and fNIRS-T (AUC = 0.734) have curves and AUC values indicating that their balance between correct and incorrect classifications in distinguishing brain signals during the word generation imagination task is inferior to TopoTempNet. This demonstrates TopoTempNet’s strong performance in classifying language imagination-related brain signals.

Figure 4c: TopoTempNet’s curve (AUC = 0.731) remains closer to the ideal top-left region across most intervals. Compared to models like fNIRS-T (AUC = 0.714) and FCS-TPNet (AUC = 0.671), their curves and AUC values show that, when handling multi-class motor imagery classification (distinguishing different motor intentions), TopoTempNet better captures complex motor imagery features and achieves more accurate classification, demonstrating a performance advantage in this complex scenario.

In summary, across all three figures, TopoTempNet’s ROC curves and AUC values lead the comparisons, indicating that for fNIRS signal classification in MA (mental arithmetic), WG (word generation imagination), and UFFT (motor imagery) tasks, it can more precisely identify target states and deliver superior classification performance compared to other models.

#### 4.1.3. Subject-Independent Evaluation

To assess the generalization ability of the TopoTempNet model on unseen subjects, this study adopts the Leave-One-Subject-Out Cross-Validation (LOSO-CV) strategy across all three datasets. In this method, during each iteration, the data from one subject are used as the test set, while the remaining data from all other subjects are used for training. This approach provides a comprehensive evaluation of the model’s adaptability and stability in handling inter-subject variability.

The process is repeated until every subject has served once as the test case. The final reported performance metrics are the average results across all subjects, aiming to objectively reflect the decoding performance and generalization ability of the model in cross-subject scenarios. The comparison results are summarized in Table 2.

### 4.2. Validation of TopoTempNet’s Capability in Extracting Brain Dynamic Patterns

#### 4.2.1. t-SNE Visualization of Learned Feature Distributions

t-SNE (t-distributed Stochastic Neighbor Embedding) [43] is a widely used nonlinear dimensionality reduction method for visualizing high-dimensional features. It preserves local structures while mapping complex features into a two-dimensional space, effectively illustrating class separability.

**Figure 5:**
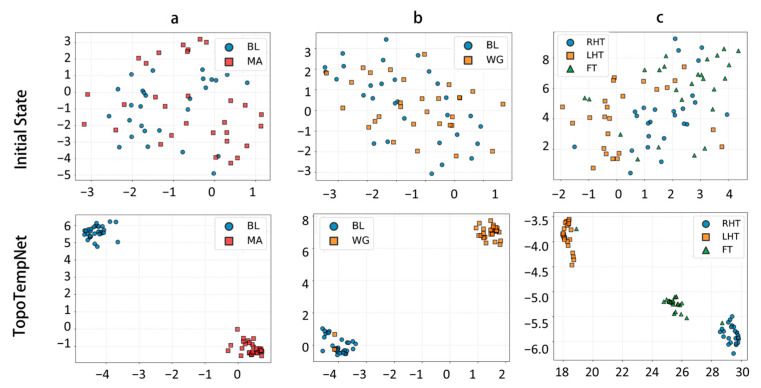
t-SNE visualizations of different datasets: (**a**) MA dataset, (**b**) WG dataset, and (**c**) UFFT dataset.

Figure 5 shows the low-dimensional representations of the MA, WG, and UFFT tasks in both the original feature space and after encoding by TopoTempNet. The results indicate that, in the original feature space, task distributions are scattered with significant class overlap and unclear boundaries. After TopoTempNet encoding, distinct clusters corresponding to different task states emerge clearly in the low-dimensional space, strongly demonstrating TopoTempNet’s effectiveness in enhancing feature discriminability and extracting key brain dynamic patterns.

#### 4.2.2. Graph Theory Feature Analysis

##### Connectivity Strength Interpretability Analysis

**Figure 6:**
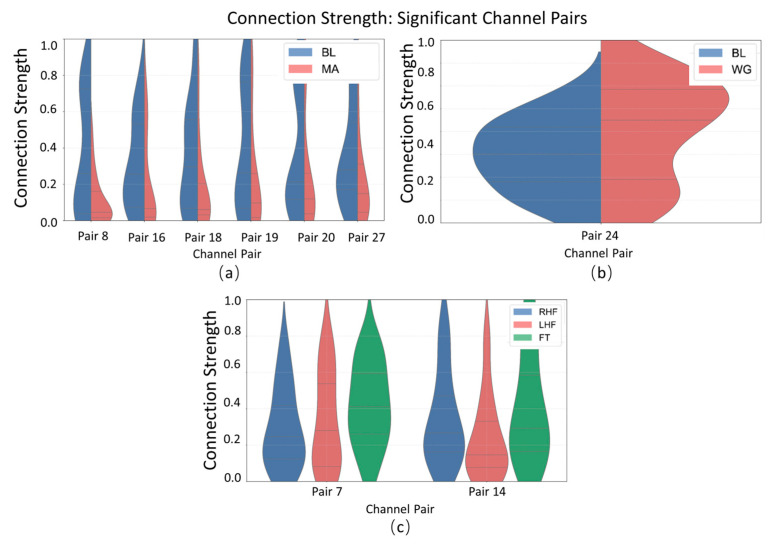
Statistical significance analysis of local channel connection strength on different datasets: (**a**) MA dataset, (**b**) WG dataset, and (**c**) UFFT dataset.

As shown in the violin plot of connectivity strength (Figure 6), distinct distribution patterns are observed across significant channel pairs in the MA (mental arithmetic), WG (word generation imagination), and UFFT (motor imagery) datasets. In certain channel pairs, the connectivity strength of the MA group is concentrated in the mid-to-high range, whereas the WG group shows a greater spread in the lower range. The UFFT group, on the other hand, exhibits unique distribution patterns in some channel pairs, differing from the other two. These findings suggest that the degree of inter-channel connectivity varies in a task-specific manner, reflecting potentially distinct neural cooperation mechanisms underlying different cognitive processes such as calculation, language generation, and motor imagery.

##### Connectivity Density Interpretability Analysis

**Figure 7:**
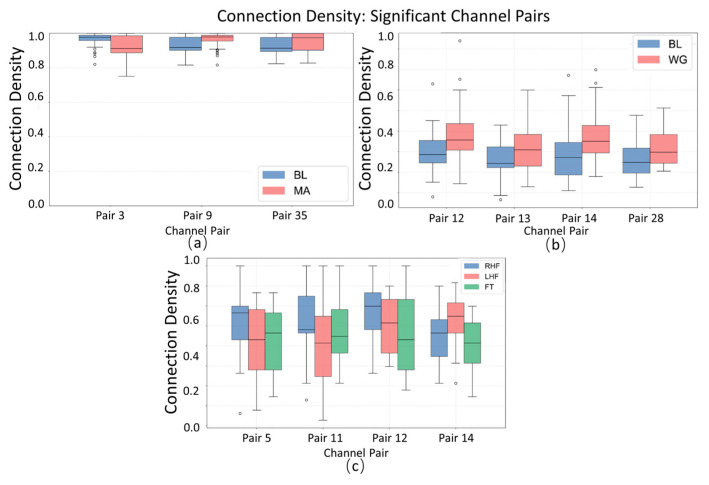
Statistical significance analysis of local channel connection density on different datasets, Circles represent outlier data points, defined as values exceeding 1.5 times the interquartile range (IQR) from the box edges in boxplot visualization.: (**a**) MA dataset, (**b**) WG dataset, and (**c**) UFFT dataset.

The boxplot of connectivity density (Figure 7) reveals the dispersion and central tendency of significant channel pairs across the MA (mental arithmetic), WG (word generation imagination), and UFFT (motor imagery) groups. In certain channel pairs, the MA group shows notable differences in median and interquartile range compared to the WG and UFFT groups, indicating a task-specific pattern of “connectivity activity frequency” in the corresponding brain regions during mental arithmetic. Additionally, the distribution of outliers varies among groups—MA exhibits more extreme high values in some channel pairs, which may suggest that the mental arithmetic task elicits statistically significant, high-frequency atypical connections in specific brain regions. Meanwhile, the density characteristics observed in the WG and UFFT groups also align with their respective task-related neural mechanisms.

##### Reciprocal of Functional Signal Mean Difference Interpretability Analysis

**Figure 8:**
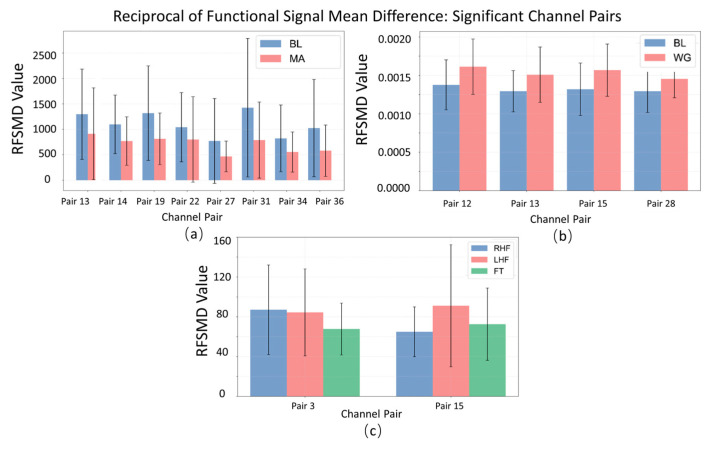
Statistical significance analysis of local channel characteristic RFSMD on different datasets: (**a**) MA dataset, (**b**) WG dataset, and (**c**) UFFT dataset.

The bar chart with error bars (Figure 8), representing the mean and standard deviation, illustrates clear differences in Reciprocal of Functional Signal Mean Difference (RFSMD) across significant channel pairs for the MA (mental arithmetic), WG (word generation imagination), and UFFT (motor imagery) groups. In certain channel pairs, the MA group shows higher average RFSMD values compared to the others, suggesting more efficient signal transmission and faster neural information integration in the corresponding brain regions during mental arithmetic. The WG group, on the other hand, exhibits lower average RFSMD values in some channel pairs, indicating that information flow may be less efficient under the word generation task, reflecting a distinct neural routing pattern. For the UFFT group, the observed RFSMD features align with the hypothesis that motor imagery requires rapid integration into motor-related regions (evidenced by relatively high RFSMD values in motor cortex-associated channel pairs), but may involve varying transmission efficiencies across different areas (reflected by uneven RFSMD distributions). Additionally, differences in the lengths of error bars between groups reflect variability in neural activity—shorter error bars in some MA group channel pairs suggest more consistent RFSMD values across individuals, indicating a relatively stable neural activation pattern during mental arithmetic.

#### 4.2.3. Neurophysiological Plausibility of TopoTempNet Features

**Figure 9:**
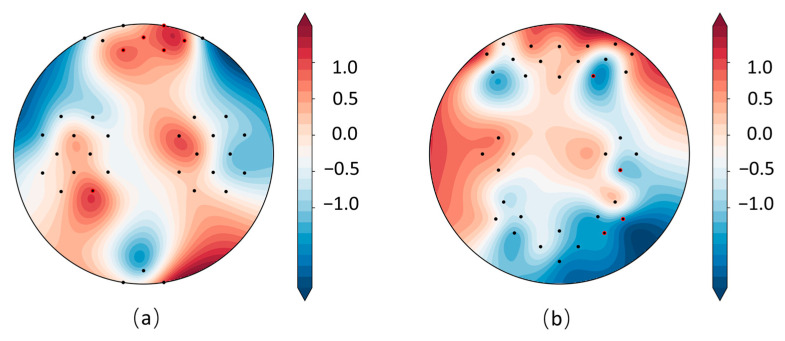
MA (**a**) and WG (**b**) dataset heatmaps, with red channels indicating significant connections (*p* < 0.05).

As shown in Figure 9, in the MA dataset (mental arithmetic task), significant channels were primarily distributed across the bilateral prefrontal cortex, encompassing both ventromedial and dorsolateral prefrontal regions (DLPFC). This distribution reflects the reliance of mental arithmetic on working memory, executive control, and numerical cognition. In contrast, in the WG dataset (word generation task), significant channels were concentrated in the right prefrontal cortex, underscoring its role in letter-guided lexical retrieval and semantic selection in coordination with left-hemisphere language areas. Overall, the observed “prefrontal–parietal” co-activation pattern captured by the model is consistent with previously reported task-related activation mechanisms [44], thereby supporting the neurophysiological plausibility of the topological features extracted by TopoTempNet. The UFFT dataset was not analyzed due to unavailable montage location information.

### 4.3. Ablation Analysis

#### 4.3.1. Module Ablation

To evaluate the effectiveness of each core module in the TopoTempNet model, ablation experiments were conducted on the three fNIRS datasets (MA, WG, UFFT) to analyze the impact of different submodules on overall performance. The ablation results are shown in Table 3, indicating that the graph theory feature module, graph attention mechanism, and bidirectional temporal modeling module each play unique yet indispensable roles across all three fNIRS datasets.

The fully integrated model combining all three modules achieved the highest accuracies of 90.04 ± 3.53, 78.33 ± 5.42, and 81.66 ± 3.23 on the MA, WG, and UFFT datasets, respectively, highlighting the synergistic benefits among these modules and providing robust support for task recognition and neural dynamic modeling.

#### 4.3.2. Ablation Study on the Effects of Global Connectivity Thresholds

The ablation experiments on global connection thresholds revealed task-specific sensitivities in both average efficiency (AE) and classification accuracy (Acc). For the mental arithmetic task, the optimal threshold was 0.3 (AE = 96.98, Acc = 90.04 ± 3.53%), with lower thresholds introducing noise (0.1: Acc = 88.43 ± 1.62%) and higher thresholds filtering out key links (0.5: Acc = 85.44 ± 4.37%). In the motor imagery task, accuracy was stable at low thresholds (0.1–0.2: 81.23–81.57%), while AE decreased as thresholds rose, with the best trade-off again at 0.3 (AE = 98.99, Acc = 81.66 ± 3.23%). The word generation task showed the highest sensitivity, with 0.3 yielding AE = 76.10 and Acc = 78.33 ± 5.42%, but performance fluctuated at lower thresholds (0.2: Acc = 83.33 ± 2.63%) and sharply declined at higher ones (0.5: Acc = 66.67 ± 8.33%). Overall, the results indicate that mental arithmetic requires precise threshold tuning, motor imagery is more robust, and word generation is highly sensitive, offering task-specific guidance for optimizing connectivity thresholds in BCI applications. The results are summarized in Table 4.

#### 4.3.3. Window Length Sensitivity Analysis

Although our framework is described as “dynamic functional connectivity,” it should be clarified that the adopted features (e.g., Pearson correlation coefficients, global efficiency) are computed within fixed sliding windows, and therefore represent a segmented quasi-static approximation of temporal variations rather than a fully continuous dynamic model as achieved by methods such as sliding-window Hidden Markov Models or time-varying state-space approaches. Our aim is not to infer latent state transitions, but to capture task-relevant fluctuations in functional connectivity within physiologically meaningful temporal segments. To delineate the reasonable boundary of this quasi-dynamic characterization, we conducted a sensitivity analysis on epoch length ($T_{epoch}$) and sliding window length (L). The results show that overly short epochs (e.g., 0.5 s) are noise-sensitive, whereas overly long ones (e.g., 3 s) smooth out critical temporal details; an intermediate epoch of 1 s provided the most stable performance across MA (fast-response), WG (variable cognitive-load), and UFFT (sustained-activation) tasks. Similarly, the optimal L range was 0.5–1 s, as shorter windows (0.2 s) were noise-dominated while longer windows (>1 s) excessively smoothed connectivity features. These findings collectively define the practical dynamic boundary of the proposed model as $T_{epoch}$ = 0.5–2 s and L = 0.5–1 s, with task-specific sensitivities such that fast-response tasks benefit from shorter segments, whereas sustained-activation tasks tolerate longer ones (see Table 5).

#### 4.3.4. Ablation of Feature Fusion Strategies

To verify the impact of scale differences in the feature fusion stage on the model’s classification performance, an ablation study was conducted to compare multiple strategies. Table 6 presents the classification accuracies (mean ± standard deviation) of different fusion methods on the MA, WG, and UFFT datasets. The results show that basic feature concatenation demonstrates a significant advantage: on the MA (90.04 ± 3.53), WG (78.33 ± 5.42), and UFFT (81.66 ± 3.23) datasets, its accuracy consistently outperforms Z-score normalized concatenation, min–max normalized concatenation, and fixed-weight concatenation. The latter three approaches, due to their forced intervention on the original feature distribution or weights, led to performance degradation on certain datasets (e.g., WG and UFFT). This indicates that the simple concatenation scheme adopted in this study, when combined with the model’s subsequent dynamic modulation mechanism, can effectively mitigate optimization bias caused by scale differences. By enabling a more natural feature integration, this strategy provides a reference for the design of feature fusion methods—namely, “lightweight intervention with reliance on module collaboration.”

### 4.4. Limitations and Future Work

Although the proposed TopoTempNet model achieved promising decoding performance on multiple public fNIRS datasets (MA, WG, UFFT), several limitations remain. The model’s applicability is currently restricted to these three task datasets, which provide only objective information (ΔHbO/ΔHbR signals, task duration, demographic data) but lack subjective assessments such as NASA-TLX scores, making it difficult to disentangle model adaptability from confounding effects of cognitive workload. Moreover, its effectiveness in more complex motor imagery scenarios or pathological populations (e.g., stroke, ADHD, ASD) has not been validated, limiting its generalizability to clinical rehabilitation. The graph-theoretic feature extraction module also involves high computational complexity, especially for global network measures, which may hinder deployment on low-power portable devices. Finally, interpretability analysis remains at the feature-visualization level without integrating neuroscientific priors to construct a refined brain network interpretation framework. Future research should therefore broaden validation to diverse tasks and clinical populations with both objective and subjective measures, adopt lightweight strategies such as knowledge distillation or sparse graph structures to reduce computational cost and enhance real-time applicability, and establish a multi-scale interpretability system linking features, brain regions, and functions to improve clinical reliability and translational potential.

## 5. Conclusions

The TopoTempNet model integrates graph-theoretic topology with hybrid temporal modeling, achieving leading decoding accuracy and strong interpretability across multiple fNIRS tasks. Experiments show that TopoTempNet significantly outperforms mainstream models such as CNN and LSTM on the MA, WG, and UFFT datasets, with subject-dependent accuracies of 90.04%, 78.33%, and 81.66%, and subject-independent accuracies of 81.06%, 78.68%, and 80.18%, demonstrating good generalization ability and stability. Kappa coefficients and AUC further confirm the model’s discriminative power and robustness.

Interpretability analysis reveals that TopoTempNet precisely captures dynamic brain features. Graph theory features significantly highlight task-specific functional connectivity patterns: increased connectivity strength in calculation-related brain regions during the MA task, enhanced connectivity density in language-related regions during the WG task, and shortened RFSMD in motor cortex during the UFFT task, reflecting task-induced brain network cooperation and optimized information transmission efficiency. These differences provide neural mechanism support for the model’s performance.

In summary, TopoTempNet excels in decoding accuracy, feature discriminability, and neural mechanism interpretation, offering strong technical support for fNIRS-BCI applications in cognitive modeling, disease diagnosis, and rehabilitation training.

## Figures and Tables

**Table 2:** LOSO-CV results for the three datasets. Bold values indicate the best performance for each evaluation metric.

Method	MA		WG		UFFT	
	Accuracy%	Kappa	Accuracy%	Kappa	Accuracy%	Kappa
CNN	70.14 ± 6.17	0.49	63.22 ± 7.30	0.32	66.16 ± 4.17	0.41
LSTM	72.17 ± 4.94	0.51	67.06 ± 4.98	0.38	66.67 ± 3.49	0.40
PVTv2-B0	76.43 ± 4.38	0.55	68.88 ± 1.92	0.40	67.02 ± 4.33	0.41
MLP-MIXer	74.28 ± 4.33	0.56	69.90 ± 7.20	0.46	68.66 ± 3.19	0.48
fNIRS-T	72.46 ± 6.12	0.51	71.26 ± 4.78	0.46	72.38 ± 4.96	0.53
FCS-TPNet	79.26 ± 5.08	0.63	78.22 ± 4.22	0.56	78.69 ± 5.38	0.56
**TopoTempNet (Ours)**	**81.06 ± 4.38**	**0.64**	**78.68 ± 4.18**	**0.56**	**80.18 ± 2.37**	**0.57**

**Table 3:** Ablation analysis of key modules in the TopoTempNet model.

Dataset	Graph-Theoretic Feature	GAM	Bi-LSTM	Accuracy%
MA WG UFFT	×	√	√	83.06 ± 4.38 72.35 ± 6.22 73.08 ± 2.67
MA WG UFFT	√	×	√	88.91 ± 3.69 77.62 ± 2.94 81.43 ± 2.44
MA WG UFFT	√	√	×	72.86 ± 3.23 64.43 ± 2.19 69.43 ± 3.72
MA WG UFFT	√	×	×	60.02 ± 9.66 58.34 ± 12.73 61.74 ± 11.38
MA WG UFFT	√	√	√	90.04 ± 3.53 78.33 ± 5.42 81.66 ± 3.23

**Table 4:** Summary of ablation results on global connection thresholds across different cognitive tasks, where AE denotes the Average Functional Connectivity Efficiency and Acc denotes the classification accuracy.

Threshold	MA		WG		UFFT	
	AE (%)	AE (%)	AE (%)	Acc (%)	AE (%)	Acc (%)
0.1	99.03	91.58	91.58	81.23 ± 2.66	99.67	81.28 ± 2.33
0.2	98.03	83.51	83.51	81.57 ± 1.68	99.33	81.23 ± 2.66
0.3	96.98	76.10	76.10	81.66 ± 3.23	98.99	81.57 ± 1.68
0.4	92.99	69.35	69.35	78.59 ± 4.36	97.26	81.66 ± 3.23
0.5	90.77	61.12	61.12	78.61 ± 3.12	95.19	78.59 ± 4.36

**Table 5:** Sensitivity analysis of window lengths: classification accuracy (Acc) under different epoch lengths ($T_{epoch}$) and sliding window lengths (L).

$T_{epoch}/s$	MA		WG		UFFT	
	L/s	Acc (%)	L/s	Acc (%)	L/s	Acc (%)
0.5	0.5	86.21 ± 4.12	0.5	75.88 ± 5.19	0.5	80.12 ± 3.57
1.0	1.0	90.04 ± 3.53	1.0	78.33 ± 5.42	1.0	81.66 ± 3.23
2.0	1.0	87.53 ± 3.89	1.0	74.62 ± 5.83	1.0	80.92 ± 2.98
3.0	1.0	84.36 ± 4.27	1.0	72.15 ± 6.01	1.0	79.35 ± 3.61

**Table 6:** Ablation results of feature fusion strategies: comparison of classification accuracy under different scale processing.

Methods	MA	WG	UFFT
Feature Concatenation	90.04 ± 3.53	78.33 ± 5.42	81.66 ± 3.23
Z-Score Normalized Concatenation	88.86 ± 4.25	78.21 ± 3.41	77.42 ± 3.29
Min–Max Normalized Concatenation	88.13 ± 4.28	72.43 ± 5.37	76.48 ± 4.73
Fixed Weighted Concatenation	89.49 ± 2.87	76.98 ± 6.21	80.16 ± 3.45

## Data Availability

The datasets used in this study are publicly available in multiple repositories. These include the Open access dataset for simultaneous EEG and NIRS brain-computerinterface (BCI) (https://doc.ml.tu-berlin.de/hBCI/). Open access NIRS dataset for classification of the unilateral finger and foot-tapping https://figshare.com/articles/dataset/Open_accessfNIRS_dataset_for_classification_of_the_unilateral_finger_and_foottapping/9783755/1 and Simultaneous acquisition of EEG and NIRS during cognitive tasks for an Open access dataset (https://doc.ml.tu-berlin.de/simultaneous_EEG_NIRS/).
